# A novel approach to simulate gene-environment interactions in complex diseases

**DOI:** 10.1186/1471-2105-11-8

**Published:** 2010-01-05

**Authors:** Roberto Amato, Michele Pinelli, Daniel D'Andrea, Gennaro Miele, Mario Nicodemi, Giancarlo Raiconi, Sergio Cocozza

**Affiliations:** 1 Gruppo Interdipartimentale di Bioinformatica e Biologia Computazionale, Università di Napoli "Federico II" - Università di Salerno, Italy; 2 Dipartimento di Scienze Fisiche, Università di Napoli "Federico II", Napoli, Italy; 3 Dipartimento di Biologia e Patologia Cellulare e Molecolare "L. Califano", Napoli, Italy; 4 INFN Sezione di Napoli, Napoli, Italy; 5 Complexity Science Center and Department of Physics, University of Warwick, Coventry, UK; 6 Dipartimento di Matematica e Informatica, Università di Salerno, Fisciano (SA), Italy

## Abstract

**Background:**

Complex diseases are multifactorial traits caused by both genetic and environmental factors. They represent the major part of human diseases and include those with largest prevalence and mortality (cancer, heart disease, obesity, etc.). Despite a large amount of information that has been collected about both genetic and environmental risk factors, there are few examples of studies on their interactions in epidemiological literature. One reason can be the incomplete knowledge of the power of statistical methods designed to search for risk factors and their interactions in these data sets. An improvement in this direction would lead to a better understanding and description of gene-environment interactions. To this aim, a possible strategy is to challenge the different statistical methods against data sets where the underlying phenomenon is completely known and fully controllable, for example simulated ones.

**Results:**

We present a mathematical approach that models gene-environment interactions. By this method it is possible to generate simulated populations having gene-environment interactions of any form, involving any number of genetic and environmental factors and also allowing non-linear interactions as epistasis. In particular, we implemented a simple version of this model in a Gene-Environment iNteraction Simulator (GENS), a tool designed to simulate case-control data sets where a one gene-one environment interaction influences the disease risk. The main aim has been to allow the input of population characteristics by using standard epidemiological measures and to implement constraints to make the simulator behaviour biologically meaningful.

**Conclusions:**

By the multi-logistic model implemented in GENS it is possible to simulate case-control samples of complex disease where gene-environment interactions influence the disease risk. The user has full control of the main characteristics of the simulated population and a Monte Carlo process allows random variability. A knowledge-based approach reduces the complexity of the mathematical model by using reasonable biological constraints and makes the simulation more understandable in biological terms. Simulated data sets can be used for the assessment of novel statistical methods or for the evaluation of the statistical power when designing a study.

## Background

Complex Diseases (CD) are caused by variations in multiple loci interacting with each other and with environmental factors [1]. Many complex traits, such as cancer, heart disease, obesity, diabetes, and many common psychiatric and neurological conditions, have large prevalence and mortality among human diseases [2,3].

The concept of Gene-Environment interaction (GxE) is theoretically central in CD [4]. It is widely accepted that GxE must be considered in CD to avoid a serious underestimation of the disease risk and inconsistencies of replication among different studies. Furthermore, taking into account the GxE could focus medical intervention by identifying sub-groups of individuals who are more susceptible to specific environmental exposures [5]. However, there are very few examples of well described GxE in scientific literature [6]. Instead, a large amount of information has been collected about both single genetic and environmental risk factors individually taken, because the majority of the studies examined the main effect of single factors instead of examining the interactions [6-8].

In our opinion, one reason for such a failure could be the statistical approach. Several statistical methods aimed at the identification of factors' interactions have been described and used to identify GxE, such as Logistic Regression [9] and Multifactor Dimensionality Reduction (MDR) [10,11]. However, the performances of these methods can be influenced by many variables such as the sample size, the number of involved factors, the type of interaction, the model of inheritance, the allelic frequencies, the distributions of the environmental factors, and the relative strength of the different factors affecting the risk of disease. Unfortunately, only some of these characteristics are known in few real populations, and therefore there is not enough information to assess the performances of statistical methods.

In this scenario, as an alternative approach, one can imagine using simulated populations in order to assess the statistical power of different methods. In population genetics, although there are several genetic data simulators (for a complete list see [12]), the vast majority have been developed to study the evolution of genomic sequences across generations (as coalescent [13] and forward-time methods [14]; for a review see [15]). Beside these tools, many others that simulate pedigrees also have been developed. They help the linkage analysis in familiar pedigrees and, hence, are useful mainly in mapping loci involved in mendelian diseases [16-22].

Regarding the modelling of the genetics role in common multifactorial complex diseases, to date, few models have been developed. The "GWAsimulator" was developed mainly to simulate pattern of linkage disequilibrium (LD) among SNPs in genome-wide studies [23]. GWAsimulator does not consider any role of the environment on the risk of disease. On the contrary, the modelling of environmental factors effect on the risk of disease is a very large field of epidemiology [24]. However, it is generally accepted that the effect of an environmental exposure on the disease risk can follow a logistic function. Indeed, the most used statistical tool for environmental factor is the logistic regression.

Among the others, two software, SIMLA [17] and QUANTO [25] are specifically designed for data sets where the disease risk is a function of interactions between genetic and environmental factors. In both models, the disease risk is based on a logistic function, where covariates are genetic factors, environmental factors and interactions. In SIMLA the data of three generations of families are simulated and the disease risk is a function of up to two genetic and two environmental factors. The user can input the relative risk associated to single factors and also combinations of any two factors. QUANTO is a tool designed to estimate the power of matched case-control, case-sib, or case-parent studies and does not actually produce simulated data sets. In QUANTO the disease risk is a function of a one gene-one environment interaction. Moreover, in QUANTO the user can input the risks associated to the environmental factor, to the genetic factor and to their interaction. SIMLA and QUANTO are valuable tools for the modelling of complex diseases, because they explicitly consider the role of GxE in disease risk. However, some limitations still exist. For example, in SIMLA it is not straightforward to simulate data of unrelated individuals as those of case-control data sets. Furthermore, the user inputs the risk associated to each factor and to each interaction of factors. In this way, after the building up of the logistic model the marginal risks that result for each single factor are not the same as those input previously. This latter can be a limitation when simulating a real dataset where only marginal risks of single factors are known, and nothing known about their relationships. Finally, these tools can describe the interactions between genetic and environmental factors only in a linear way and they are not easily extensible to more complex interactions.

We propose a novel method, the Multi-Logistic Model, that mathematically describes gene-environment interactions that are similar to those found in case-control studies. By this method it is possible to model GxE in any form, involving any number of genetic and environmental factors, also allowing gene-gene interactions, as epistasis. A simple version has been implemented in the Gene-Environment iNteraction Simulator (GENS), designed to simulate case-control data sets where a one gene-one environment interaction influences the disease risk. Moreover, to make easier the simulation of data nearer to those from previous studies or literature we used common epidemiological measures as input. This also makes the tool friendlier to the biomedical community.

## Results

### The Multi-Logistic Model for gene-environment interaction

The mathematical approach behind the simulation of the disease risk involving GxE is based on a system of logistic relationships. We called this approach Multi-Logistic Model (MLM) and specifically designed it to describe disease risk in data sets that simulate case-control samples. In the simulated data sets, each individual has *G *genetic factors and is exposed to *E *environmental factors. Genetic factors are denoted by  where *a *= 1, ..., *G*. The genetic factors are biallelic Single Nucleotide Polymorphisms which result in three diploid genotypes, namely the first homozygote (AA, *i*_*a *_= 1), the heterozygote (Aa, *i*_*a *_= 2) and the second homozygote (aa, *i*_*a *_= 3). Genetic frequencies for each factor are denoted by *P*^*G*^() where ∀*a *. The environmental variables, instead, are denoted by , where *b *= 1, ..., *E *and *j*_*b *_is an index which runs over the possible discretized values of the variable *b*. They are characterized by exposure probabilities denoted by *P*^*E*^() (where again ∀*b *). It is worth noticing we preferred to present the mathematical description concerning a discrete environmental variable only in order to keep it simpler. However, the model is more general and can be referred to as both continuous or discrete variables.

**Figure 1:**
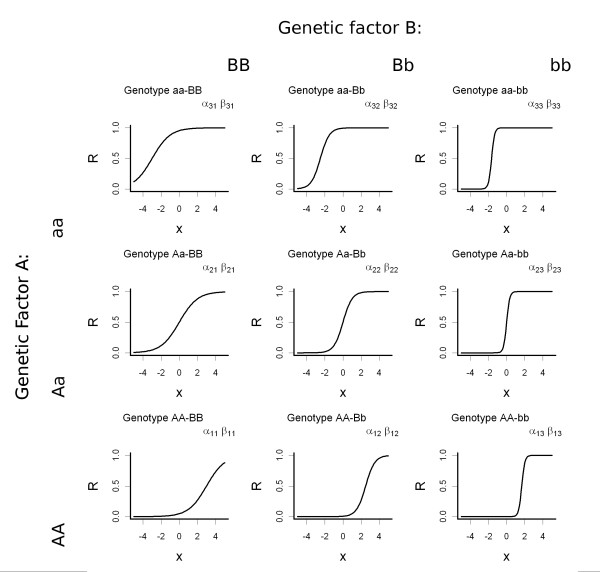
**Multi-logistic model applied to a two genetic-one environmental factors condition**. On the y-axis is reported the disease risk (*R*) and on the x-axis is reported the level of exposure of the environmental factor. The relationship is modelled by the Eq. 2. For each combination of genetic factors there are different *α*_*i *_and *β*_*i *_that models the relationship between environmental exposure and disease risk.

Let us consider a particular individual characterized by (*E *+ *G*) values of  and . In general the disease risk *R *is a function of all of them. The disease risk for such an individual () is defined by the conditioned probability(1)

where *P *(affected| ) is the probability of the individual to be *affected*. In our model we assume a logistic expression for *R*:(2)

where  and  are free parameters determined by the genetic factors and governing the shape of the function. Figure 1 shows an example of the model in case of 2 genetic and 1 environmental factor interacting.

### Gene-Environment iNteraction Simulator

We implemented the MLM in the Gene-Environment iNteraction Simulator (GENS). For the sake of simplicity we describe, in this phase, a simple interaction between one genetic and one environmental factor even though we continue to describe an individual by assigning to him a (*E *+ *G*)-tuple of characteristics. As a consequence of this choice, the MLM gets a simpler form. In particular, we can drop the indexes *a *and *b *in the expression of disease risk (2). Thus, by denoting with *g*_*i *_the genotype of the chosen gene and with *x*_*j *_the exposure level of the environmental factor involved, we have(3)

In other words, the MLM reduces to three logistic functions, one for each genotype.

It is possible to think of *α*_*i *_as the basal genetic disease risk in individuals with that genotype. The greater is *α*_*i *_the stronger is the disease risk, independently of the contribution of the environmental factor. In particular, for vanishing *α*_*i *_there is no basal risk and the risk is totally ascribed to the environmental exposure (*x*_*j*_). Analogously, *β*_*i *_represents the coefficient associated to the environmental exposure, thus the greater is *β*_*i *_the greater risk is associated to an increasement in the environmental exposure. In other words, *β*_*i *_models, for genotype *i*, the susceptibility to the environmental factor exposure. Consequently, for vanishing *β*_*i *_the environmental exposure has no effect on the disease risk.

To describe populations by standard epidemiological measures, we implied the relative risk as the measure of the role of a genetic factor on the disease risk. In particular, by defining the Total Risk (*T R*) in a specific genotype *i *as(4)

(which holds under the hypothesis of independence among different environmental variables) one can define the Relative Risk *RR*_*kl *_≡ *T R*_*k*_/*T R*_*l*_.

We take one homozygote as a reference point (say AA, denoted with *i *= 1), the other homozygote (say aa, *i *= 3) has an equal or larger risk than the first one, and the heterozygote (Aa, *i *= 2) has a risk ranging within the two homozygotes. Furthermore, we assume the relative risk of heterozygote to be within those of the two homozygotes (1 ≤ *RR*_21 _≤ *RR*_31_). In particular, if the heterozygote risk is the same of the first homozygote a recessive effect is simulated. If the heterozygote has the same risk of the second homozygote a dominant effect is simulated. Other situations are called co-dominant.

Formally, the relative risk of heterozygote *RR*_21 _is defined as(5)

where the *W *allows to model various inheritance effects: recessive (*W *= 0), dominant (*W *= 1), and co-dominant (0 <*RR*_21 _< 1) [17].

Marginal risk of the environmental factor is input as the odds ratio of the increase of one unit in the level of exposure. This value is then transformed in the coefficients *β*_*i *_of the multi-logistic model. Anyway, at most only one *β*_*i *_is provided by the user, leaving the tool deriving other values to respect all the constraints.

#### Type of GxE interaction

**Figure 2:**
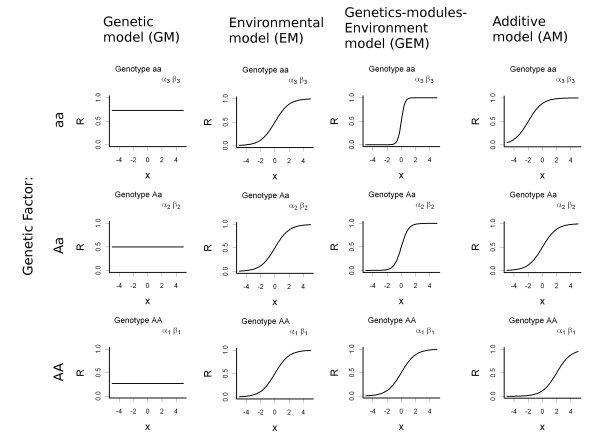
**Type of GxE interactions modeled by KAPS**. On the y-axis is reported the disease risk (*R*) and on the x-axis is reported the level of exposure of the environmental factor. The relationship is modelled by the Eq. 3. For each combination of genetic factors there are different *α*_*i *_and *β*_*i *_that follows the specific constraints (Table 1). In the Environmental Model (EM), the disease risk is dependent only by the environmental exposure level, thus the environment-risk relationship is the same across genotype (same slope and no shift). In the Genetic Model (GM), the disease risk depends on genetic factor only, thus the environment has no role on the disease risk (the curve is flat) while the risk is different across genotypes (height of the curve). In the third model (AM), the disease risk depends on both genetic and environmental factors; the relationship between environmental exposure and disease risk is the same in each genotype (same slope), but in each genotype there is a different basal risk (shift). In the fourth model (GEM), the genetic factor influences the relationship between environmental exposure and disease risk (slope). However, there is no different basal genetic risk (no shift).

To describe the GxE in biological understandable terms, we consider a genetic only and an environmental only model and two models of interactions that involve both genetic and environmental factors (Table 1 and Figure 2). The first two models could be useful as reference.

**Table 1:** Relationships among the coefficients of the Multi-Logistic Model and the type of interaction.

Interaction model	Constraints
Genetic Model	*α*_1 _≤ *α*_2 _≤ *α*_3 _and *β*_1 _= *β*_2 _= *β*_3 _= 0

Environmental Model	*α*_1 _= *α*_2 _= *α*_3 _and *β*_1 _= *β*_2 _= *β*_3 _= *β *≠ 0

Gene Environment interaction Model	*α*_1 _= *α*_2 _= *α*_3 _and *β*_1 _≤ *β*_2 _≤ *β*_3_

Additive Model	*α*_1 _≤ *α*_2 _≤ *α*_3 _and *β*_1 _= *β*_2 _= *β*_3 _= *β *≠ 0

Type of gene-environment interactions are expressed as constraints among coefficients of the Multi-Logistic Model. This approach allows to specify the type of interaction to simulate in a simple manner. Another interesting consequence is that for each type of interaction only a subset of coefficients needs to be specified.

In the first model, the *Genetic Model *(GM), each individual carrying a genotype has the same disease risk regardless of the environmental exposure. This situation is modelled by giving a vanishing effect to the environmental variable, namely fixing all the *β*_*i *_equal to zero. In the second model, the *Environmental Model *(EM), the risk is due to the environmental exposure only. This situation is modelled by imposing *α*_*i *_and *β*_*i *_equal across the genotypes with a non-vanishing *β*_*i*_. This choice provides the same risk independently of the carried genotype.

The third model simulates the scenario where the *gene modules response to environment *(Gene Environment interaction Model - GEM). In this case the genetics do not directly affect the disease risk, but modules the response to the environmental exposure. In other words, some genotypes are more prone than others to develop the disease if exposed to the same environmental level. In this interaction model all the *α*_*i *_are equal (no direct genetic effect) while *β*_*i *_are different. The last is the *Additive Model *(AM), where genetic and the environment influence the risk directly, independently and additively. Moreover the environmental exposure has the same effect in all the genotypes (equal *β*_*i*_). For this model, there are no complex interactions between the genetics and the environmental exposure. However, the risk is the sum of that due to the genetic predisposition and that due to the environmental role. Of course the user can create further types of GxE by freely imposing *α*_*i *_and *β*_*i*_.

#### Knowledge-Aided Parametrization System

To translate the population parameters into coefficients of the MLM, we implemented the Knowledge-Aided Parametrization System (KAPS). This module derives the values of *α*_*i *_and *β*_*i *_starting from genotype frequencies, relative risk and model of inheritance of the genetic factor, distribution and odds ratio of the environmental factor, type of GxE and the proportion of affected individuals in the sample (*m*).

The key issue is that the overall disease frequency in the population *m *is given by(6)

Dividing Eq. 6 by *T R*_1 _and by means of some algebraic manipulation, it is straightforward to show that(7)

In a similar way it is possible to derive the expressions for the marginal risks of the other genotypes. By numerically solving this set of three equations (one for each *T R*_*i*_) it is possible to obtain *a*_*i *_and *β*_*i *_coefficients that match at most the user's requests.

#### Algorithm and Implementation

**Figure 3:**
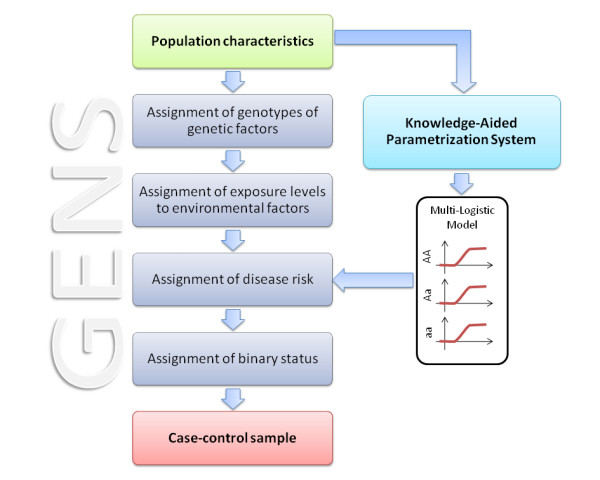
**Flowchart of GENS**. Starting from desired population characteristics, GENS assigns to each individual the genotypes of genetic factors and the exposure levels of environmental factors. Beside, the module KAPS uses population characteristics to compute coefficients of the Multi-Logistic Model. Thus, on the basis of individual characteristics and Multi-Logistic Model, the individual disease risk is computed. The last step is the assignment of disease status to individuals (affected/not affected) according to their disease risks.

The simulation procedure is divided into several steps (Figure 3). First of all, the genotypes of *G *genetic factors and the levels of exposure of *E *environmental factors are assigned to the *N *individuals.

Consequently, the sample population characteristics (Table 2) are input by the user and hence the coefficients of the MLM are calculated. Finally, the disease risk and the disease status are assigned to each individual.

**Table 2:** Parameters required by GENS. Description of parameters required by GENS in order to produce a simulated case-control sample. These parameters are translated into coefficients for the Multi-Logistic Model by the Knowledge-Aided Parametrization System.

Parameter	Description
*N*	Number of individuals

*G*	Number of genetic factors

*E*	Number of environmental factors

*P*^*G*^()	Frequency of genotype *i*_*a *_of genetic factor *g*^*a *^(*a *= 1, ..., *G*)

*P*^*E*^()	Exposure probabilities, where *b *= 1, ..., *E *and *j*_*b *_is an index which runs over the possible discretized values of the variable *b*

*m*	Overall disease frequency in the population

TypeOfGxe	Type of GxE: Genetic (GM), Environmental (EM), Gene Environment interaction (GEM, Additive (AM)

*RR*_31_	Relative risk of high-risk homozygote

*W*	Model of inheritance: recessive (*W *= 0), dominant (*W *= 1), co-dominant (0 <*W *< 1)

*β*	Odds ratio of the disease risk of an individual exposed to *x*_*j *_with respect to one exposed to *x*_*j *_+ 1

Concerning the genetic factors, the user can provide the allelic frequencies or allow the simulator to randomly select them (with a uniform distribution between 0.1 and 0.9). In both cases, the Hardy-Weinberg's law is used for the calculation of the frequencies of the genotypes. Afterwards, by means of a Monte Carlo method, the genotype of each genetic factor is randomly assigned to each individual according to the genotypic frequencies. Similarly for the environmental factors, the user can use a distribution function, among a set of predefined ones, or provide an empirical distribution *P*^*E*^(). Again by a Monte Carlo process the exposures of environmental factors are assigned according to distribution functions.

After the assignment of the genetic and environmental factors to individuals, the next step is the assignment of the phenotype. For this process the system computes the coefficients of the MLM in order to create the relationship between population characteristics, type of GxE interaction, and disease risk. The actual computation of the coefficients is performed by KAPS that, by means of Eq. 7 and similar ones for *T R*_2 _and *T R*_3_, solves numerically the resulting system of three equations and returns *α*_1_, *α*_2_, *α*_3_, *β*_1_, *β*_2 _and *β*_3_.

The disease risk (0 ≤ *R*(*g*_*i*_, *x*_*j*_) ≤ 1) is assigned by the MLM (Eq. 3) by using the parameters previously identified. In particular, for each individual his genotype *i *establishes the coefficients *α*_*i *_and *β*_*i *_computed by KAPS, while the exposure level is the value of the covariate *x*_*j*_. The last step is to assign a disease status (affected/not affected) to the individuals. Again by a Monte Carlo process, the system generates a random number with uniform distribution in [0, 1] and assigns to the individual the status 1 (affected) if this number is less then his risk *R*(*g*_*i*_, *x*_*j*_), or 0 (not affected) if otherwise.

An implementation of GENS is freely available on Sourceforge https://sourceforge.net/projects/gensim as a set of Matlab 7.0 scripts that can be freely modified to address different requirements (different risk function, multiloci interaction, etc.).

## Discussion and Conclusions

In this article we present a novel mathematical approach to model GxE in complex diseases. This approach is based on a Multi-Logistic Model (MLM) and it is specifically tailored to model disease risk in data set that simulates case-control samples. We implemented this method in Gene-Environment iNteraction Simulator (GENS), a tool designed to yield case-control samples for GxE. These tools could be useful to generate simulated data sets in order to assess the performances of statistical methods.

The necessity to provide simulated populations is due to the difficulty of obtaining real populations in which enough parameters are known to be related to the phenotype. Furthermore, during the design of a statistical study, simulated populations can also be used to estimate the expected statistical power when assuming different types of GxE [26]. We focused on inputing characteristics extracted by real populations (such as allelic frequencies, environmental factor distributions, risk given by genetic and environmental factors, etc.). In this way it is also easy to replicate real populations and evaluate the change of statistical power due to changes of the parameters as the sample size (*N*) and the disease frequency (*m*) and the type of GxE and etc.

The key idea underlying the MLM is the modelling of the disease risk in each combination of genetic factors (genotypes) as a different mathematical function of the environmental exposures (Figure 1). In this way it is possible to model any type of interaction between genetic and environmental factors, also complex and non-linear ones. We based our approach on the logistic function. This function is widely used in epidemiological studies and has several advantages. It follows the Weber-Fechner law and as the value of the risk factor increases it naturally ranges from 0 to 1 [27]. Moreover, the coefficients of the covariates correspond to the logarithm of the odds ratio due to a one-unit increase (in this case the environmental factor) [27]. In particular, in order to calculate the disease risk, the genetic factors of individuals sets coefficients of the function while the environmental factors assign a value to its covariates.

We implemented the MLM in the Gene-Environment iNteraction Simulator, a GxE simulator for case-control studies. The intended audience of GENS is the biomedical community, thus the main efforts have been to describe populations by standard epidemiological measures, to implement constraints to make the simulator behaviour biologically meaningful, and to define the GxE in biological understandable terms. In theory, the MLM can model interactions of multi-genetic and multi-environmental factors. However, for the sake of simplicity we focused on an interaction between one genetic and one environmental factor. In this way it is much easier to use as input standard epidemiological measures. Nevertheless, even in this simple situation, the handling of the interaction is not straightforward. Furthermore, in simulated populations besides the involved factors there are other ones that act as noisy background, as frequently occurs in real data sets.

Even in this simple scenario, modelling the desired characteristics of a population can be very difficult, except for some particular and simple cases, mainly because it is necessary to provide several coefficients to the mathematical model. However, having several coefficients with a difficult interpretation is a common pitfall when modelling complex interactions. Therefore, to overcome this limitation we have implemented the Knowledge-Aided Parametrization Subsystem (KAPS). This system exploits a set of reasonable biological constraints to reduce the complexity of the system. First of all, concerning the genetic factors, we imposed that the risk assigned to the heterozygote falls between the two homozygotes. Secondly, we adopted a *qualitative *description of the GxE. In particular, each type of GxE can be modelled as a set of equality and inequality of *α*_*i *_and *β*_*i *_among genotypes. We pre-determined two types of GxE, an additive (AM) and a modulative type (GEM). The user has only to select which type of GxE must be simulated, without providing additional information. In this way, we can reduce the complexity of the system and, therefore, reduce the degrees of freedom of the mathematical model. Finally, KAPS solves the system of equations to derive coefficients in order to comply with both biological constraints and population characteristics imposed by the user. As a consequence, to simulate a population only classical epidemiological parameters have to be provided (Table 2). However, the user can simulate any kind of interaction by the freedom of inputing all the coefficients of the MLM, and even to substitute the logistic expression with a different one.

In population genetics, data simulation has been mainly used to study population evolution, linkage disequilibrium, and pedigree of mendelian disease [16-22]. Although some very interesting tools have been specifically designed for complex diseases [17,25], some limitations still exist. For example they do not directly produce case-control data sets. GENS is specifically designed to produce case-control data sets as close as possible to real ones in a simple manner. In addition, differently from a *naive *logistic model, the MLM allows modelling non-liner phenomena such as epistasis.

One of the shortcomings of GENS compared to other tools could be the limitation of one gene-one environment interactions. However, this choice has been made because it is easier to describe and understand the joint and single role of the factors. It should be noted that this limitation accounts mainly to the present implementation, in particular to KAPS. In fact, the multi-logistic model can be easily used to simulate multi genetic-multi environmental factor interactions by applying Eq. 2 and providing enough coefficients. The number of environmental factors are increased by adding additional covariates in the functions to consider their effects. Instead, the number of genetic factors involved in the disease risk is increased by defining additional logistic functions in the multi-logistic model. For example, with the software a file is provided containing parameters of a non-linear interaction among three genetic and two environmental factors. Furthermore, the multi-logistic model can be extended to use different functions for each combination of genetic factors.

As our approach is widely based on a Monte Carlo process, the system naturally takes into account the randomness present in any real data sets obeying to probabilistic laws. In other words, data sets created with the same characteristic results to be randomly different.

In conclusion, by the multi-logistic model and GENS it is possible to simulate case-control samples of complex diseases where gene-environment interactions influence the disease risk. The user has full control of the main characteristics of the simulated populations and the Monte Carlo process allows random variability. A knowledge-based approach reduces the complexity of the mathematical model by using reasonable biological constraints and makes the simulation more understandable in biological terms. Simulated data sets can be used for the assessment of novel statistical methods or for the evaluation of statistical power when designing a study.

## Authors' contributions

RA, MP and GM conceived and developed the model. RA, DDA and GR implemented the scripts. MP and SC curated the biological aspect. GM, MN, GR and SC participated in the design and coordination of the overall study, and drafted the manuscript. All authors read and approved the final manuscript.
